# Structure and optical properties of a trimetallic cobalt–molybdenum–sodium metal–organic framework

**DOI:** 10.1107/S205322962600077X

**Published:** 2026-02-09

**Authors:** Benjamin J. Moore, Jeremiah P. Tidey, Craig I. Hiley, Richard I. Walton

**Affiliations:** a Department of Chemistry, University of Warwick, Coventry, CV4 7AL, United Kingdom; b Department of Physics, University of Warwick, Coventry, CV4 7AL, United Kingdom; Hong Kong University of Science and Technology, Hong Kong

**Keywords:** 3D ED, metal–organic framework, coordination polymer, solvothermal, multivariate, crystal structure

## Abstract

The crystallization of a trimetallic cobalt–molybdenum–sodium metal–organic <!?tlsb=-0.05pt>framework, poly[μ-ben­zene-1,3,5-tri­car­box­yl­ato-tetra-μ-ox­ido-co­balt­mol­yb­de­num­tri­so­dium], UOW-10 or [Na_3_Co(MoO_4_)(BTC)]_*n*_, is achieved by solvothermal synthesis using benzene-1,3,5-tri­carb­oxy­lic acid (H_3_BTC, C_9_H_6_O_6_) as a ligand precursor, Na_2_MoO_4_·2H_2_O and Co(NO_3_)_2_·2H_2_O as metal sources, and *N*,*N*-di­methyl­formamide (DMF) as the solvent. 3D electron diffraction (3D ED) reveals that the structure crystallizes in the monoclinic space group *P*2_1_/*c*, with lattice parameters of *a* = 9.718 (2), *b* = 18.250 (3), *c* = 6.892 (9) Å, α = γ = 90, β = 96.156 (15)°, *V* = 1214.7 (4) Å^3^ and *Z* = 4. The phase purity of the bulk sample was confirmed using syn­chro­tron powder X-ray diffraction. The organic ligands form a 2D layer, where cobalt and molybdenum are found, with sodium cations located between the layers. There are four crystallographically distinct sodium sites: three exhibit a distorted octa­hedral coordination geometry, while the remaining site is seven-coordinate. The cobalt has trigonal bipyramidal coordination geometry and molybdenum exhibits a tetra­hedral coordination geometry. Half the sodium cations in the structure forms 1D column-like motifs *via* shared oxygen edges along the crystallographic *c* axis, which are cross-linked in *b* by the cobalt and molybdenum sites *via* bridging O atoms, while the other half of the sodium cations form 2D ribbons in the *ac* plane, propagating along *c*, linked by sharing oxygen edges and faces. The optical properties of UOW-10 were investigated through the use of UV–Vis spectroscopy, showing a bandgap of 1.8 eV. Deconvolution of the features in the visible-light region reveals that four peaks are present, which can all be ascribed to the *d–d* transitions from the trigonal bipyramidal cobalt. By means of thermogravimetric analysis (TGA) and variable-tem­per­a­ture powder X-ray diffraction (VT-PXRD), it is demonstrated that the material has thermal stability to 410 °C, after which structure collapse occurs, leading to a mixture of Na_2_MoO_4_, CoO and Co_3_Mo above 900 °C.

## Abstract 2

The structure of a trimetallic MOF synthesized under solvothermal conditions has been determined using 3D electron diffraction with bulk sample purity assessed by Rietveld analysis of high-resolution powder X-ray diffraction. The 3D connected structure con­tains tetra­hedral Mo^VI^, five-coordinate Co^II^ and six- and seven-coordinate Na, connected by benzene-1,3,5-tri­carboxyl­ate, and shows a UV–Vis spectrum consistent with the trigonal bipyramidal cobalt(II) centre.

## Introduction

In the past 30 years, metal–organic frameworks (MOFs), crystalline materials with potential porosity (Batten *et al.*, 2012 ▸) constructed from inorganic building blocks (termed secondary building units, SBUs) and organic ligands, have gained attention for their application in areas including catalysis (Wei *et al.*, 2020 ▸; Dhakshinamoorthy *et al.*, 2018 ▸; Farrusseng *et al.*, 2009 ▸), gas adsorption (Li *et al.*, 2009 ▸; Han *et al.*, 2019 ▸; Xiao *et al.*, 2025 ▸), sensing (Koo *et al.*, 2019 ▸), proton conductivity (Sharma *et al.*, 2023 ▸; Chen *et al.*, 2023 ▸) and drug delivery (Lawson *et al.*, 2021 ▸). One of the distinct advantages of MOFs in com­parison to other porous materials such as zeolites is their high degree of tunability (Hao *et al.*, 2018 ▸). Systematic alterations to the metal nodes of the material or the organic linkers in the structure can result in varied properties. For example, in the field of photocatalysis, functionalizing organic linkers with auxochromes such as amino, hydroxyl and aldehyde groups has been shown to be a powerful tool to modulate the absorption edge of MOFs, thereby improving photocatalytic performance (Sun *et al.*, 2013 ▸; Chambers *et al.*, 2017 ▸). In the field of gas adsorption, decoration of organic linkers with amino groups has become a common strategy to increase CO_2_ adsorption capacity (Kim *et al.*, 2020 ▸).

Recently, multivariate metal–organic frameworks (MTV MOFs) have attracted attention as a means of further modulating the properties of the materials (Helal *et al.*, 2017 ▸). MTV MOFs can generally be defined as MOFs which are constructed by multiple organic ligands and/or more than one metal. This enhanced degree of tailorability has proved important in achieving high-performing materials (Castells-Gil *et al.*, 2020 ▸; Yuan *et al.*, 2016 ▸).

With regards to synthesis, MTV MOFs can be prepared either by direct crystallization under solvothermal conditions or by post-synthesis exchange of either cations or ligands. The former is exemplified by MOF-74 which can be prepared with up to ten individual metals incorporated in a one-step solvothermal synthesis, albeit with some heterogeneity in their distribution from crystal to crystal (Wang *et al.*, 2014 ▸). The latter approach may avoid the possibility of phase separation during synthesis into a mixture of crystalline phases. For example, the synthesis of mixed-linker systems, such as PCN-701, PCN-702 and PCN-703, could not be achieved from direct synthesis, but instead could only be accessed *via* a ligand installation approach (Yuan *et al.*, 2015 ▸). Here, the exploitation of the free hydroxyl groups in the low-connectivity Zr_6_ cluster allows for the precise installation of two different carboxyl­ate linkers of varying lengths, thereby increasing the connectivity of the metal cluster from eight to 12 (Yuan *et al.*, 2016 ▸).

With regards to mixed-metal systems, post-synthetic approaches have also been reported. For example, in the UiO-67–bpy system, the free N atoms in the 2,2′-bi­pyridine-5,5′-di­carboxyl­ate linker can be used as sites to chelate various metals, thereby generating novel multivariate MOFs (Maza *et al.*, 2015 ▸). Another common approach is the utilization of preformed heterometallic clusters that are used as metal nodes in the construction of the MOF. For example, in PCN-415, a pre-formed [Ti_8_Zr_2_O_12_(COO)_16_] is utilized as a building block to construct a novel photoactive MOF (Yuan *et al.*, 2017 ▸).

In this article, a heterometallic metal–organic framework from a readily available organic ligand is reported by a direct solvothermal reaction. The synthesis strategy was to use metal cations with distinct chemical nature. Co^II^ can be viewed as a borderline Lewis acid due to its moderate charge density, while Mo^VI^ is a hard Lewis acid due to its high charge density. The third metal, sodium, is more ionic in character, with little preferrence for coordination geometry. This difference in hardness may be inter­esting for applications which can benefit from mixed Lewis acidity, such as the field of catalysis (Castells-Gil *et al.*, 2020 ▸). The optical properties of the new material were measured and are strongly influenced by the cobalt coordination geometry, and thermogravimetric analysis and variable-tem­per­a­ture powder X-ray diffraction show the material to have good thermal stability.

## Experimental

### Synthesis of Na_3_Co(MoO_4_)BTC (UOW-10)

Co(NO_3_)_2_·2H_2_O (87.3 mg, 0.6 mmol), Na_2_MoO_4_·2H_2_O (72.5 mg, 0.6 mmol) and benzene-1,3,5-tricarboxylic acid (H_3_BTC; 126 mg, 1.2 mmol) were added to a 25 ml Teflon-lined stainless steel autoclave, followed by 10 ml of DMF. The mixture was stirred for 1 h, then sealed and heated at 175 °C for 17 h, followed by cooling to room tem­per­a­ture over a period of 72 h. The resulting solid was filtered *via* a Büchner funnel and washed by pouring DMF (3 × 15 ml) over the sample, followed by drying at 70 °C in air.

### Single-crystal 3D electron diffraction

Crystal data, data collection and structure refinement details are summarized in Table 1 ▸. For the 3D ED experiment, the sample was ground lightly between glass slides and dispersed dry onto a copper-supported amorphous carbon TEM grid. This was loaded at room tem­per­a­ture *via* a JEOL high-tilt specimen holder into a Rigaku XtaLAB Synergy-ED electron diffractometer, operated at 200 kV and equipped with a Rigaku HyPix-ED hybrid pixel array area detector. Data were collected at 293 (5) K as single-rotation scans using the *CrysAlis PRO* system (Rigaku OD, 2025 ▸) for multiple crystallites appearing as microcrystalline chips using continuous rotation electron diffraction with a selected area aperture of 2 µm apparent diameter. No indication of beam damage was observed. Although data consistently indexed to the reported cell, the sample exhibited ubiquitous nonmerohedral twinning by twofold rotation about the *c* axis and, as a consequence, datasets could not be merged; data are pre­sent­ed for the most com­plete and high quality of these collections. The diffraction pattern and an image of the sample studied on the TEM grid can be seen in Fig. S1.

Data were indexed and integrated with scaling and an empirical absorption correction was applied using *CrysAlisPRO*. The structures were solved using *SHELXT* (Sheldrick, 2015 ▸) and refined using *olex2.refine* in the kinematic approximation, applying an extinction correction to broadly account for dynamical scattering, as implemented in *OLEX2* (Bourhis *et al.*, 2015 ▸; Dolomanov *et al.*, 2009 ▸), using published scattering factors (Saha *et al.*, 2022 ▸).

H atoms are placed geometrically and have positions and isotropic displacement parameters riding on their parent atoms at neutron average distances (Allen & Bruno, 2010 ▸). Non-H atoms were refined with anisotropic atomic displacement parameters and global rigid-bond restraints were employed to improve the physical sense of the displacement parameters. Experimental and refinement information are con­tained within the deposited CIF along with structure factors and an embedded .res file.

### Bulk sample characterization

Powder X-ray diffraction data for phase identification were recorded on a Siemens D5000 X-ray diffractometer, using a Cu *K*α_1/2_ source producing 1.5418 Å wavelength X-rays. *In situ* powder X-ray diffraction was performed using a Bruker D8 instrument with Cu *K*α_1/2_ radiation, fitted with an Anton Paar XRK 900 chamber and a VÅNTEC solid-state detector. Samples were heated to 900 °C in flowing air and diffraction patterns were recorded at inter­vals of 20 °C. Room-tem­per­a­ture high-resolution syn­chro­tron powder X-ray diffraction data were collected at Beamline I11 at Diamond Light Source [λ = 0.8249705 (3) Å] for structural analysis of the bulk powder. The diffraction pattern of UOW-10 was fitted to using *TOPAS 6*, with lattice parameters being the only sample parameters refined *via* the Rietvield method using the crystallographic model obtained by 3D ED (Coelho, 2018 ▸). Thermogravimetric analysis was performed using a Mettler–Toledo TGA/DSC1 under an air atmosphere with a heating rate of 5 °C. IR spectroscopy was performed on a Bruker ALPHA FT–IR ATR spectrometer, with spectra collected in the range 650–4000 cm^−1^. Diffuse-reflectance UV–Vis spectroscopy was conducted on a Shimadzu UV-2600 spectrophotometer in the range 200–800 nm. Scanning electron microscopy (SEM) images were collected using a Zeiss SUPRA 55VP FEGSEM instrument.

## Results and discussion

### Crystal structure of UOW-10

**Figure 1:**
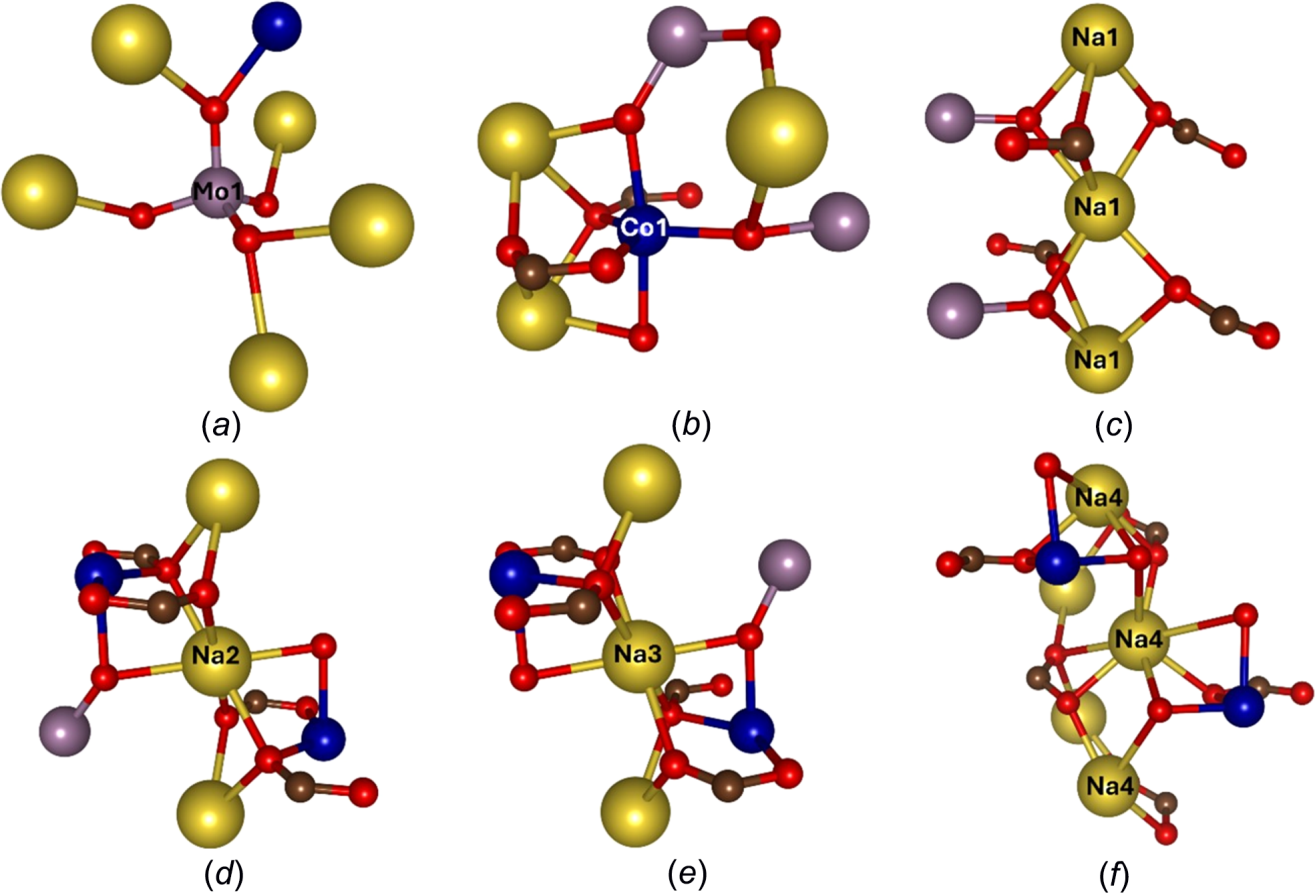
Local coordination environment of the atoms in UOW-10, with Co atoms shown in dark blue, Na atoms shown in yellow, Mo atoms shown in purple and O and C atoms shown in red and brown, respectively. (*a*) The Mo coordination geometry and connectivity, (*b*) the Co coordination geometry and connectivity, and (*c*)–(*f*) the Na1–Na4 coordination environments.

The material crystallized at a synthesis tem­per­a­ture in the range 150–200 °C, as indicated by the formation of purple microcrystals, with a powder X-ray diffraction pattern consistent with the structure determination (see following). The resulting crystals were too small for laboratory or syn­chro­tron single-crystal X-ray diffraction (Fig. S2 in the supporting information), and so 3D ED was used to solve the structure of the material. This revealed the crystals to be a heterometallic metal–organic framework con­taining sodium, cobalt and molybdenum. The material, CoMoNa_3_O_4_(BTC), crystallizes in the monoclinic space group *P*2_1_/*c*, with lattice parameters *a* = 9.718 (2), *b* = 18.250 (3), *c* = 6.892 (9) Å, α = γ = 90, β = 96.156 (15)°, *V* = 1214.7 (4) Å^3^ and *Z* = 4. Lists of bond lengths and angles can be found in Tables S1 and S2, respectively. The molybdenum forms in a four-coordinated tetra­hedral geometry and is not coordinated to the BTC linker but instead by edge-sharing oxides with cobalt and sodium [Fig. 1 ▸(*a*)]. The cobalt centres are five-coordinate with two of the equatorially coordinated O atoms resulting from a η^1^-coordination of the BTC linker [Fig. 1 ▸(*b*)] and the others being oxide anions shared between cobalt, molybdenum and sodium. The Co—O bond lengths range from 1.976 (7) to 2.101 (10) Å, which is typical for Co^II^ MOFs according to the Inorganic Crystal Structure Database (ICSD, https://icsd.products.fiz-karlsruhe.de/) and the Cambridge Structural Database (CSD; Groom *et al.*, 2016 ▸). Indeed, bond valence sums suggest the oxidation state of cobalt is Co^II^ (Table S3) as opposed to Co^III^ (Brown & Altermatt, 1985 ▸).

There are four, fully occupied, crystallographically independent sodium sites, two of which are located at inversion centres in the structure. The Na1, Na2 and Na3 sites are six-coordinate, and all adopt a distorted octa­hedral coordination geometry. In contrast, the Na4 site is seven-coordinate. While the local connectivity of these sites vary, for all Na sites, four of the coordinating O atoms are provided by the carboxyl­ate groups, while the remaining O atoms are oxides shared between neighbouring metal centres. For the Na1 site [Fig. 1 ▸(*c*)], the remaining oxides are shared with a molybdenum centre, for the Na2 and Na3 sites [Figs. 1 ▸(*d*) and 1(*e*)], the remaining oxides are shared with both a molybdenum and cobalt centre, and at the Na4 site, the remaining [Fig. 1 ▸(*f*)] oxides are shared between two distinct symmetry-equivalent cobalt centres.

**Figure 2:**
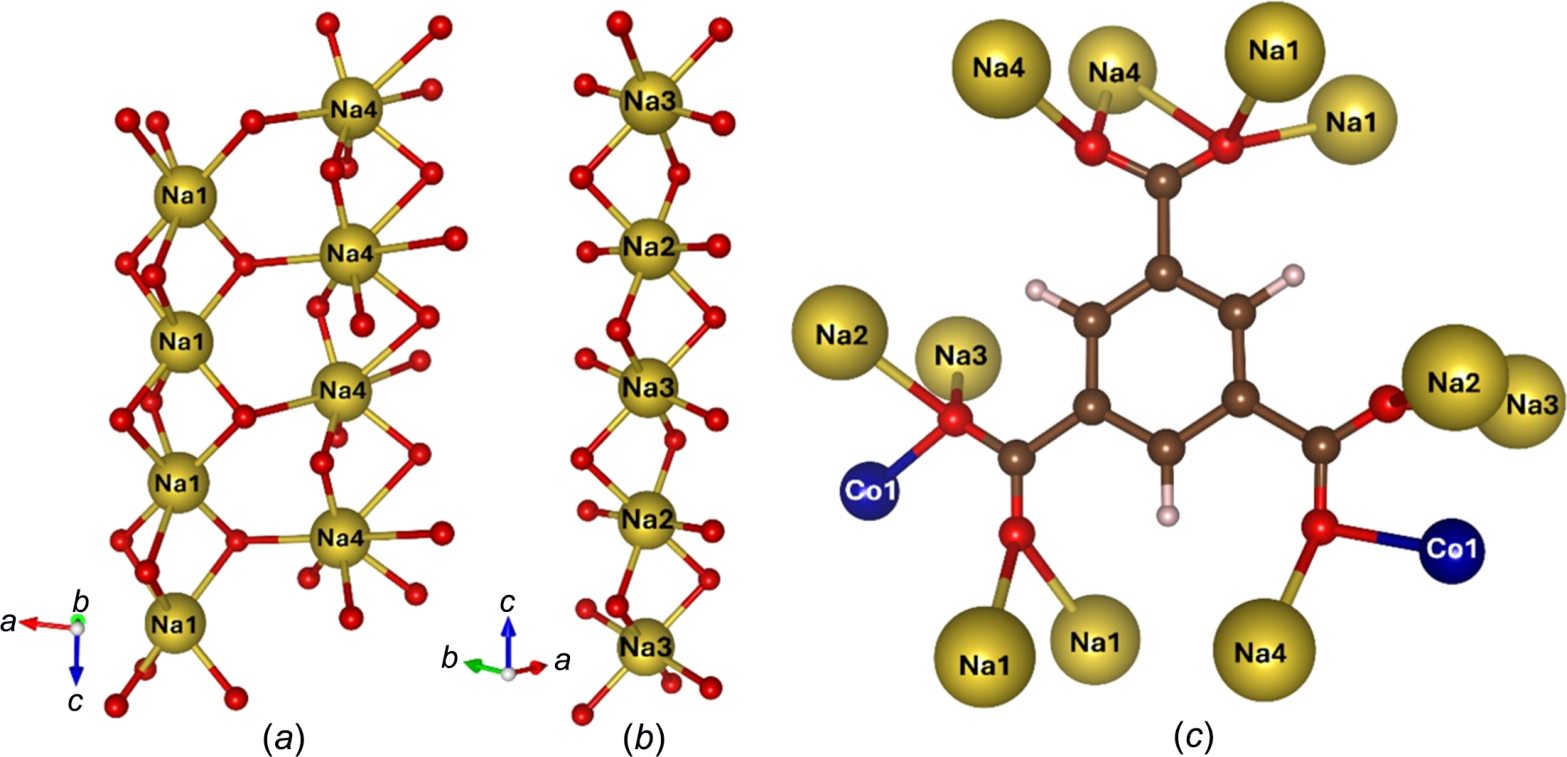
The structural features of UOW-10, with Co atoms shown in dark blue, Na atoms shown in yellow and O, C and H atoms shown in red, brown and white, respectively. (*a*) The Na1 and Na4 double-column ribbon running along the crystallographic *c* axis, (*b*) the Na2/3 column running along the crystallographic *c* axis and (*c*) the connectivity of the BTC ligand in UOW-10.

The Na1-centred octa­hedra face-share with one another, thereby forming a column running along the crystallographic *c* axis. Another column running along the crystallographic *c* axis is formed by edge-sharing Na4 atoms. These two columns are inter­connected by a μ_3_-OCO group from the carboxyl­ate moiety to give a double column or ribbon-like feature [Fig. 2 ▸(*a*)]. The Na2 and Na3 octa­hedra share edges with one another, also forming a column along the crystallographic *c* axis [Fig. 2 ▸(*b*)]. The Na2/Na3 column and Na1/Na4 double column are cross-linked by the cobalt and molybdenum sites, respectively. With regards to bond lengths, the Na—O bonds vary between 2.202 (13) and 2.908 (12) Å, which falls in the typical range for Na—O bonds according to the ICSD and CSD.

The organic ligand coordinates to several metal ions giving a com­plex coordination mode of the carboxyl­ate groups [Fig. 2 ▸(*c*)]. One of the carboxyl­ate groups binds in a μ_4_:η^1^:η^2^:η^1^:η^1^ fashion to four Na sites. Another carboxyl­ate group binds in a μ_4_:η^1^:η^1^:η^1^:η^1^ fashion to three Na sites and one Co site. The final carboxyl­ate group binds in a μ_5_:η^1^:η^1^:η^1^:η^1^:η^1^ fashion to four Na sites and one Co site.

**Figure 3:**
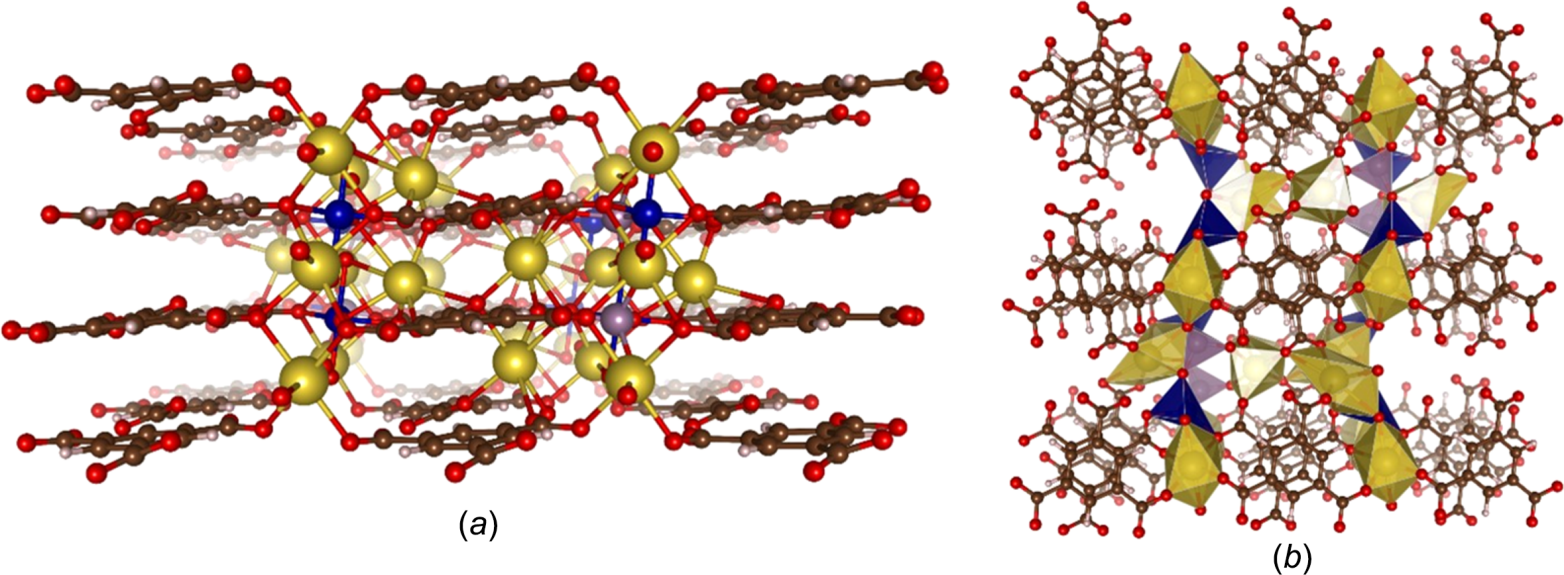
Extended views of UOW-10, with Co atoms shown in dark blue, Na atoms shown in yellow, Mo shown in purple and O, C and H atoms shown in red, brown and white, respectively. (*a*) View of the structure along the crystallographic *b* axis, revealing the sodium columns separated by Co and Mo atoms. (*b*) View of the structure along the crystallographic *c* axis. Na, Co and Mo polyhedra are shown in gold, blue and purple, respectively.

Figs. 3 ▸(*a*) and 3(*b*) depict an extended views of the structure along the crystallographic *b* and *c* axes, respectively. In Fig. 3 ▸(*a*), it can be observed that all the BTC linkers are coplanar with each other connected by the Co centres, so forming layers. No free carb­oxy­lic acid groups are observed in the structure, which is in agreement with FT–IR data which show no characteristic bands of the non-ionized carboxyl groups of H_3_BTC (νOH 3082 cm^−1^; νC=O 1720 cm^−1^). Instead, bands in the ranges 1541–1613 and 1354–1433 cm^−1^ are present, which correspond to the asymmetric COO^−^ and symmetric COO^−^ vibrations, respectively (Fig. S3) (Maiti *et al.*, 2015 ▸). Sodium cations are located between the 2D layers, while the cobalt and molybdenum cations are approximately in the plane of the BTC linkers.

As seen in Fig. S4, com­paring the IR spectra of UOW-10 and Na_2_MoO_4_·2H_2_O in the range 1000–825 cm^−1^, the region corresponding to Mo=O and Mo—O stretches, shows close agreement, thereby providing further evidence of the MoO_4_ tetra­hedral units in the structure. The Mo—O bond lengths vary between 1.699 (10) and 1.731 (7) Å, which is similar to what is seen in other molybdates (Chithambararaj *et al.*, 2013 ▸). Bond valence sums (Table S4) indicate that the oxidation state of molybdenum is Mo^VI^ (Chen *et al.*, 2002 ▸).

One of the more inter­esting features of the structure of UOW-10 is the presence of a five-coordinate Co^2+^ metal centre. In UOW-10, the trigonal bipyramidal coordination geometry of the cobalt is achieved as a consequence of edge-sharing O atoms with two sodium sites, as well as corner-sharing with a third sodium and one molybdenum site. This coordination environment would prevent the coordination of, for example, additional solvent mol­ecules which would otherwise allow the system to achieve octa­hedral geometry. Although rare, trigonal bipyramidal geometries have been observed in mol­ecular com­plexes. These cases typically involve the use of bulky chelating ligands to stabilize the geometry. Among inorganic materials, the five-coordinate CoO_5_ configuration is relatively rare, with only 138 crystal structures reported in the ICSD, com­pared to 1624 for CoO_6_ and 232 for CoO_4_. This trend is consistent with the CSD where there are 4397 CoO_6_ structures, 551 CoO_4_ structures and 360 CoO_5_ structures. Among the CoO_5_-con­taining com­pounds many incorporate tetra­hedral structural units – including molybdates, sulfates, phosphates, vanadates and arsenates (Engel *et al.*, 2009 ▸; Lü *et al.*, 2014 ▸; Baies *et al.*, 2006 ▸; Smith Pellizzeri *et al.*, 2020 ▸; de Pedro *et al.*, 2010 ▸). This could indicate that tetra­hedral structural units can help stabilize lower cobalt coordination geometries, as seen in UOW-10.

Usually, cobalt-con­taining MOFs con­tain the metal in sites that are either four- or six-coordinate. For example, in cobalt-based zeolitic imidazolate frameworks (ZIFs), the cobalt cation is typically coordinated by four imidazolate ligands to afford tetra­hedrally coordinated metal centres (Park *et al.*, 2006 ▸). In contrast, in cobalt-based Hofmann clathrates, cobalt is six-coordinate, with four cyano groups coordinating in equatorial positions and nitro­gen-con­taining ligands in axial positions (Pei *et al.*, 2022 ▸). Notably, cobalt MOFs can reversibly transform between six- and four-coordinate structures; for example, in PCN-224(Co), where the cobalt metal centres in the porphyrin core are six-coordinate (four equatorial N atoms and two axial solvent mol­ecules), upon activation the solvent mol­ecules can be removed and the coordination number reduced to four to yield a square-planar Co^II^ metal centre.

Cobalt-based MOFs can also reversibly transform from six- to five-coordinate structures to generate square-pyramidal coordination geometry. This is more commonly seen in triazolate-based MOFs such as MAF-X27, MIT-20 and Co-BTTri (Liao *et al.*, 2015 ▸; Park *et al.*, 2017 ▸; Xiao *et al.*, 2016 ▸). There are some carboxyl­ate-based systems which can also generate this coordination geometry. For example, MOF-74(Co) (also known as CPO-27) (Rosi *et al.*, 2005 ▸; Dietzel *et al.*, 2008 ▸) and its isorecticular analogues (Peng *et al.*, 2020 ▸; Ahmed *et al.*, 2024 ▸) once dehydrated show a square-pyramidal coordination geometry. Cobalt MOFs with trigonal bipyramidal coordination geometries have been reported previously, usually ap­pearing in mixed-ligand systems that con­tain both carboxyl­ate and nitro­gen-based donors (Geng *et al.*, 2012 ▸). In cases where this coordination geometry is solely observed with carboxyl­ate-based donors, the inorganic building unit is usually a chain or cluster as opposed to a monometallic metal node (Kim *et al.*, 2012 ▸; Wang *et al.*, 2013 ▸; Sarma *et al.*, 2012 ▸).

**Figure 4:**
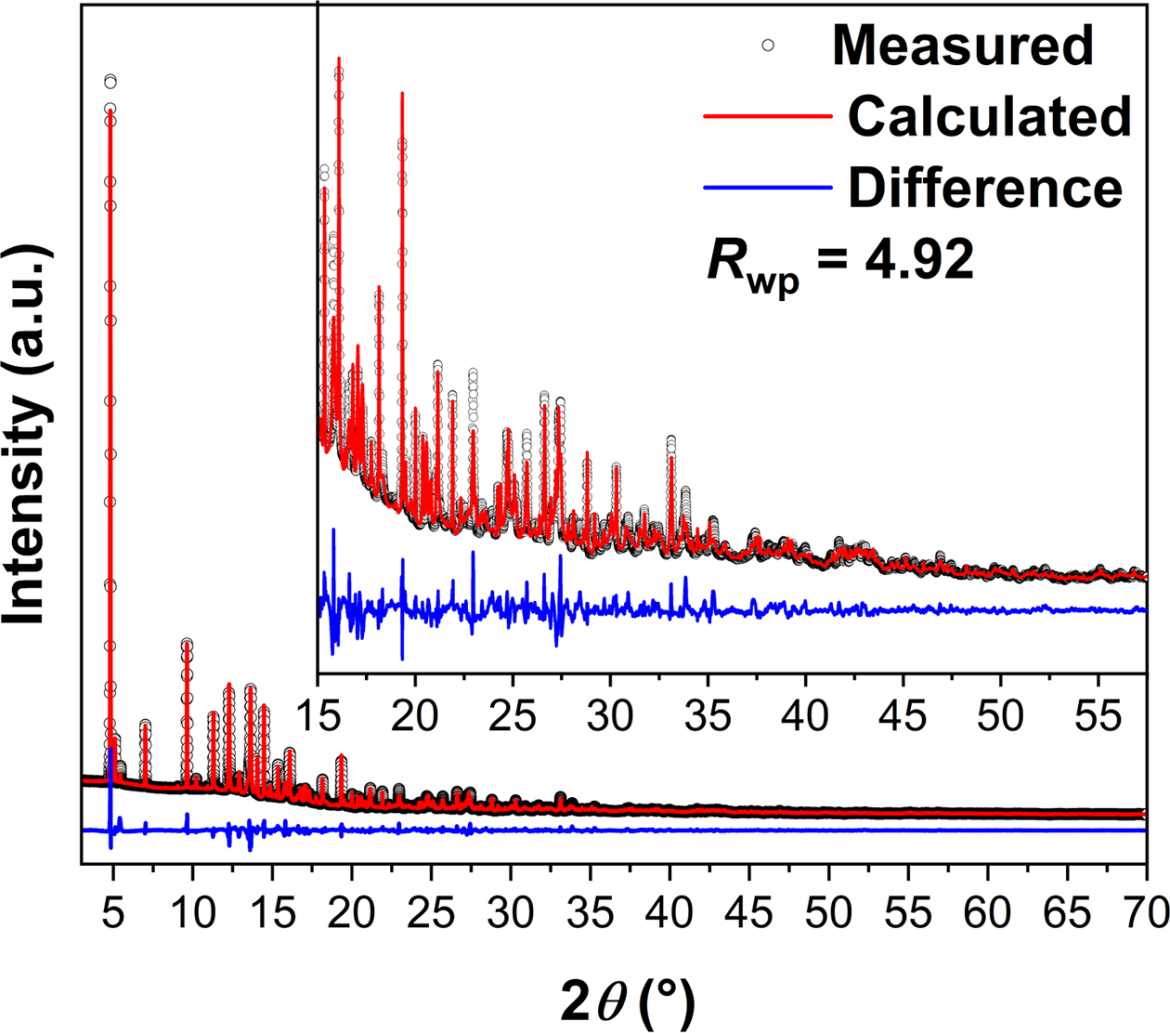
Final Rietveld fit for UOW-10 using the 3D ED structure against syn­chro­tron powder X-ray diffraction data [λ = 0.8249705 (3) Å]. See text for refined lattice parameters.

In order to verify the structural model from the 3D ED and to verify the purity of the bulk sample, high-resolution syn­chro­tron PXRD data were collected. As seen in Fig. 4 ▸, the Rietveld fit (*R*_wp_ = 4.92%) confirms that the structural model is representative of the bulk material and the sample is of high purity since no other diffraction peaks are observed. The refined lattice parameters [*a* = 9.88678 (11), *b* = 18.53948 (15), *c* = 6.99808 (9) Å, α = γ = 90 and β = 96.382 (5)°] are in good agreement with those obtained from 3D ED [*a* = 9.718 (2), *b* = 18.250 (3), *c* = 6.892 (9) Å, α = γ = 90 and β = 96.156 (15)°].

### Optical properties

**Figure 5:**
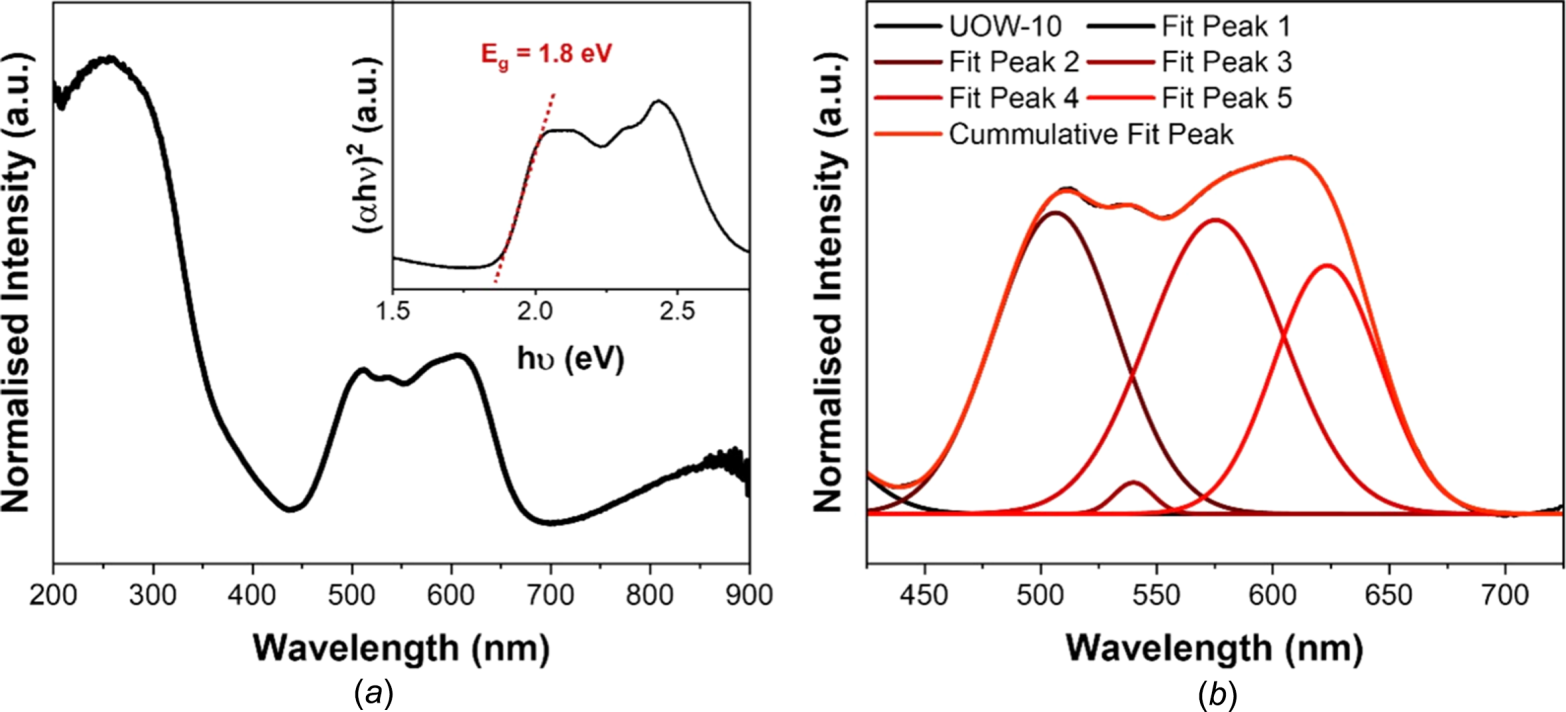
Optical properties of UOW-10. (*a*) UV–Vis diffuse reflectance spectrum of UOW-10 demonstrating that the sample absorbs in the UV and visible regions. The inset is a Tauc plot constructed from the UV–Vis data, indicating the existence of a band gap at 1.8 eV. (*b*) Deconvolution of the visible-light region of the UV–Vis spectrum.

The UV–Vis diffuse reflectance spectrum [Fig. 5 ▸(*a*)] shows the existence of two main features, one centred in the UV region and the other in the visible-light region. The first feature appears in the range 200–300 nm and is very similar to that seen in the acid form of the ligand, H_3_BTC, with a slight red shift. This feature most likely corresponds to π–π* transitions within the BTC ligand, in good agreement with what has been observed previously in the literature for other BTC-based MOFs (Wang *et al.*, 2015 ▸). The feature seen in the visible-light region in the range 450–650 nm is more structured. Deconvolution of this region reveals that four peaks make up the signal [Fig. 5 ▸(*b*) and Table S5]. Inter­estingly, the UV–Vis spectrum of UOW-10 shares similarities with that of a mixed-ligand cobalt MOF, [Co(C_12_H_8_N_2_)(HO_3_P–C_2_H_4_–PO_3_H)], reported by Fu *et al.* (2007 ▸). This is presumably a consequence of cobalt also being in a trigonal bipyramidal coordination geometry in this structure. In their work, several transitions can be seen in the visible-light region at λ_max_ = 504, 534 and 624 nm. In UOW-10, four transitions can be seen in a similar region, *i.e.* λ_max_ = 506, 540, 575 and 623 nm. The transitions at 506, 575 and 623 nm can be attributed to *d*–*d* transitions from a trigonal bipyramidal crystal field of the cobalt metal centre (Companion & Komarynsky, 1964 ▸). Pre­sumably the weak transition observed at 540 nm, which cannot be accounted for by crystal field splitting, is a spin-forbidden or vibronically allowed *d*–*d* transition. The corresponding Tauc plot [Fig. 5 ▸(*a*), inset] indicates that the material shows a bandgap of ∼1.8 eV.

### Thermal stability of UOW-10

**Figure 6:**
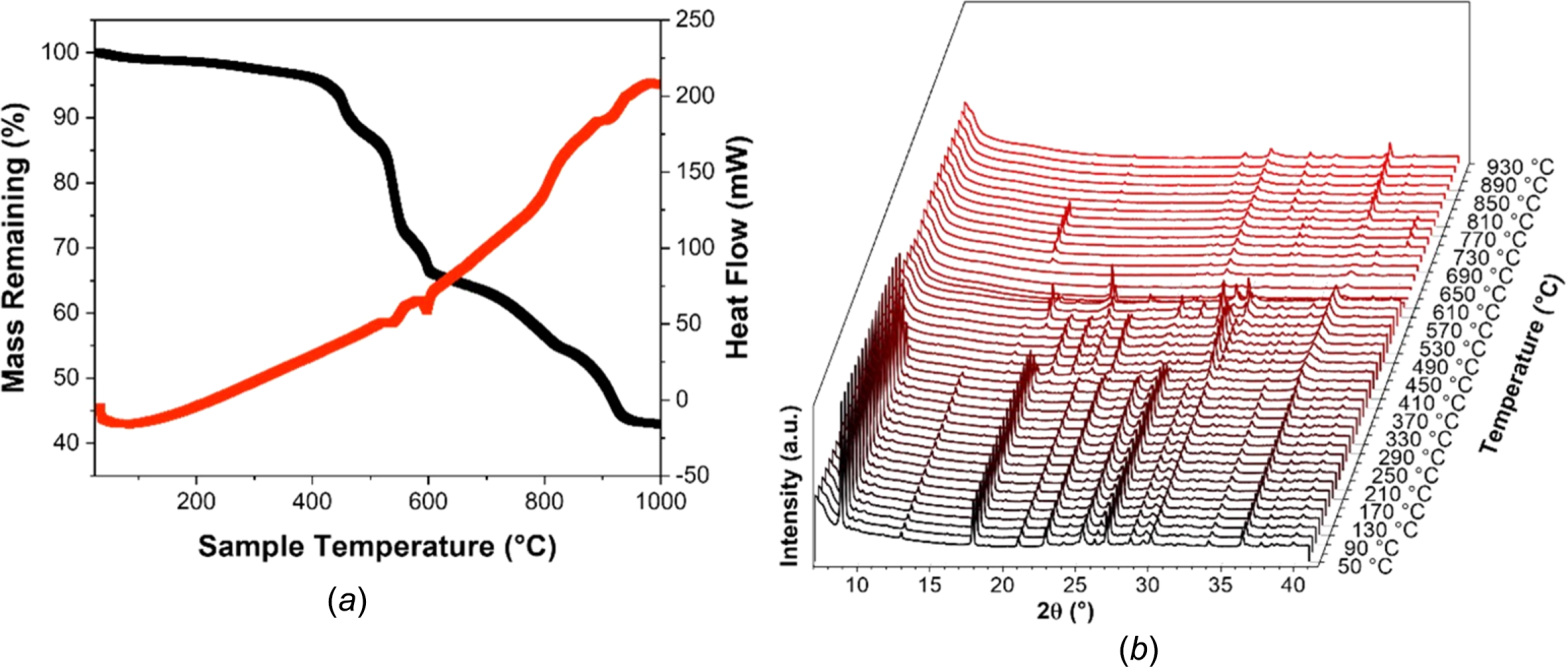
Assessing the thermal stability of UOW-10. (*a*) Thermogravimetric analysis (TGA) data collected from 50 to 1000 °C and (*b*) variable-tem­per­a­ture powder X-ray diffraction (VT-PXRD) of UOW-10 demonstrating the good thermal stability of the material and the formation of several phases upon structure collapse at tem­per­a­tures greater than 410 °C.

In order to gain insight into the thermal stability of UOW-10, a combination of variable-tem­per­a­ture *in-situ* PXRD (VT-PXRD) and thermogravimetric analysis (TGA) were used. As seen in Fig. 6 ▸(*a*), an initial small loss in mass (<3%) is observed in the range 35–410 °C. As there are no solvent mol­ecules present in the crystal structure, this suggests this mass loss corresponds to the removal of surface solvent mol­ecules. This is corroborated by VT-PXRD [Fig. 6 ▸(*b*)], which revealed that in this tem­per­a­ture range, crystallinity is maintained and the diffraction intensity remains constant. The VT-PXRD reveals that above 410 °C the framework begins to collapse as Bragg peaks associated with UOW-10 begin to decrease in intensity, while new peaks associated with another phase begin to increase in intensity. This result is corroborated by the TGA which shows the onset of a significant mass loss. Between 430 and 920 °C, PXRD shows several distinct changes which implies that several inter­mediate crystalline phases form in the decom­position process. This is also observed in the TGA, where there are several mass-loss features. Although the identity of the inter­mediate phases could not be identified, the Bragg peaks observed appear at higher angles than initially present in UOW-10, indicating that the inter­mediate materials have smaller unit cells, and likely correspond to dense phases. After heating at 930 °C, the remaining material can be assigned as being a mixture that con­tains Na_2_MoO_4_, CoO and Co_3_Mo (Fig. S5).

## Conclusion

A novel trimetallic cobalt-, molybdenum- and sodium-con­taining metal–organic framework constructed using a commercially available organic ligand, benzene-1,3,5-tri­carboxyl­ate, was crystallized directly under hydro­thermal conditions. The material presents a unique structure which unusually con­tains five-coordinate Co^2+^ connected to both Na and Mo *via* shared O atoms. Although this leads to a rather dense structure, the characterization of this phase suggests that other heterometallic MOFs may be possible with unique structures, if more extended linkers were to be used.

## Supplementary Material

Crystal structure: contains datablock(s) I, global. DOI: 10.1107/S205322962600077X/qf3071sup1.cif

Structure factors: contains datablock(s) I. DOI: 10.1107/S205322962600077X/qf3071Isup2.hkl

Images of crystals, IR spectra, powder XRD, tables of bond distances and angles, deconvolution of UV-Vis spectra and bond valence sums. DOI: 10.1107/S205322962600077X/qf3071sup3.pdf

CCDC reference: 2449088

## Figures and Tables

**Table 1:** 3D ED experimental details

Crystal data	
Chemical formula	[Na_3_Co{MoO_4_}(C_9_H_3_O_6_)]
*M*_r_	494.96
Crystal system, space group	Monoclinic, *P*2_1_/*c*
Temperature (K)	293 (5)
*a*, *b*, *c* (Å)	9.718 (2), 18.250 (3), 6.8892 (9)
β (°)	96.156 (15)
*V* (Å^3^)	1214.7 (4)
*Z*	4
Radiation type	Electron, λ = 0.0251 Å
Collection method	Continuous rotation electron diffraction (cRED)
Probe details	2 µm diameter selected area aperture
μ (mm^−1^)	Not calculated

Data collection	
Diffractometer	Rigaku XtaLAB Synergy-ED HyPix-ED, electron source at 200 keV
No. of measured, independent and observed [*I* ≥ 2σ(*I*)] reflections	3862, 3862, 3183
*R*_int_	0.181
(sin θ/λ)_max_ (Å^−1^)	0.626

Refinement	
*R*[*F*^2^ > 2σ(*F*^2^)], *wR*(*F*^2^), *S*	0.182, 0.406, 1.04
No. of reflections	3862
No. of parameters	222
No. of restraints	108
H-atom treatment	H-atom parameters constrained
Δφ_max_, Δφ_min_ (e Å^−1^)	1.67, −1.54

Computer programs: *CrysAlis PRO* (Rigaku OD, 2025 ▸), *SHELXT* (Sheldrick, 2015 ▸), *olex2.refine* (Bourhis *et al.*, 2015 ▸) and *OLEX2* (Dolomanov *et al.*, 2009 ▸).
