# Supplementary material for: Structure and optical properties of a trimetallic cobalt–molybdenum–sodium metal–organic framework
Source: Acta Crystallogr C Struct Chem. 2026 Feb 9;82(Pt 3):102–9. doi: 10.1107/S205322962600077X (PMC12961748; doi:10.1107/S205322962600077X)
Supplement: Supplementary file 3 [file c-82-00102-sup3.pdf]

Supporting information

Structure and optical properties of a trimetallic cobalt-molybdenum-sodium metal-organic framework

Benjamin J Moore, Jeremiah P. Tidey, Craig I. Hiley and Richard. I. Walton

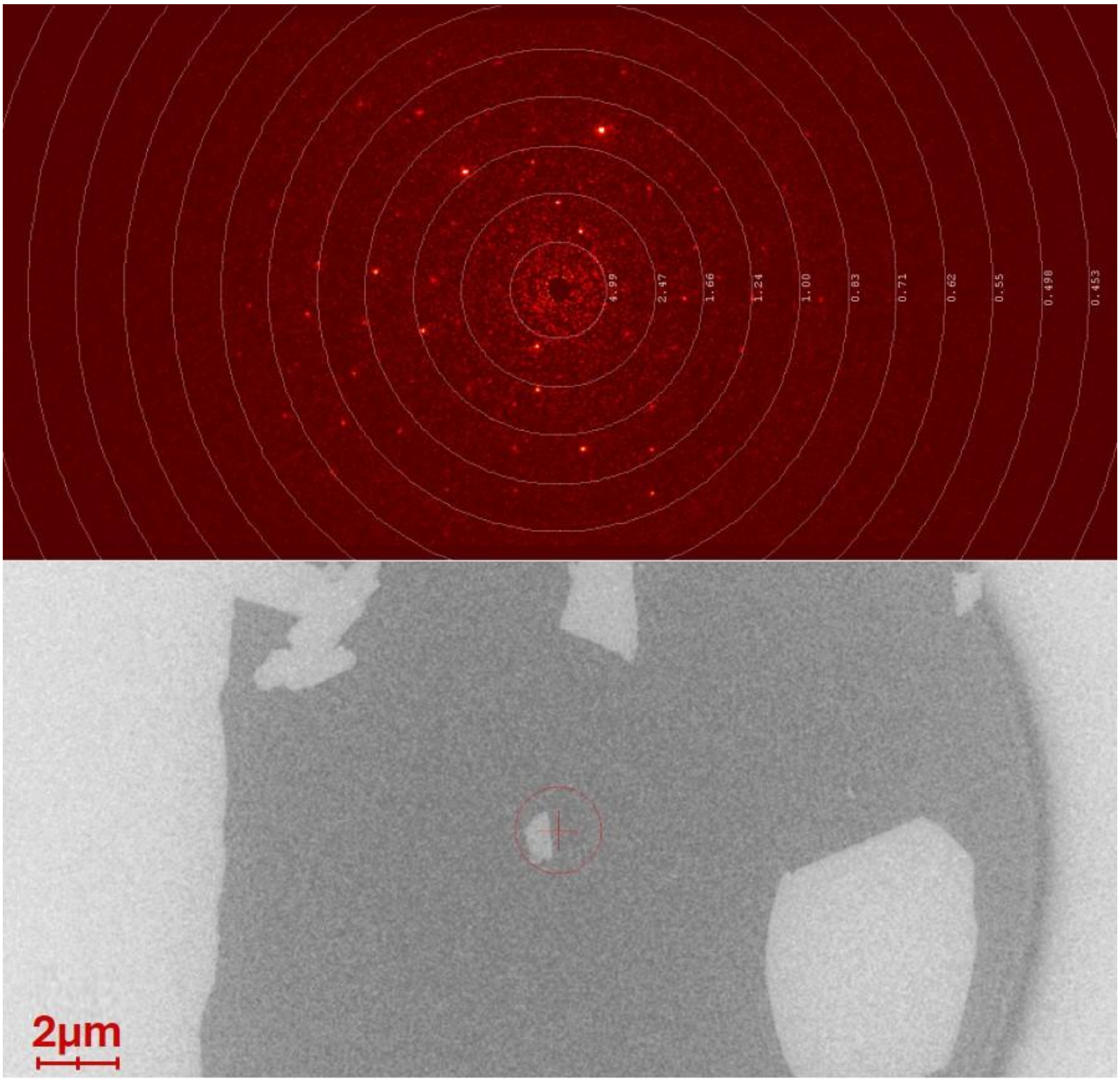

Figure S1 3D ED diffraction pattern of UOW-10 (top) and image of sample studied on the TEM grid (bottom)

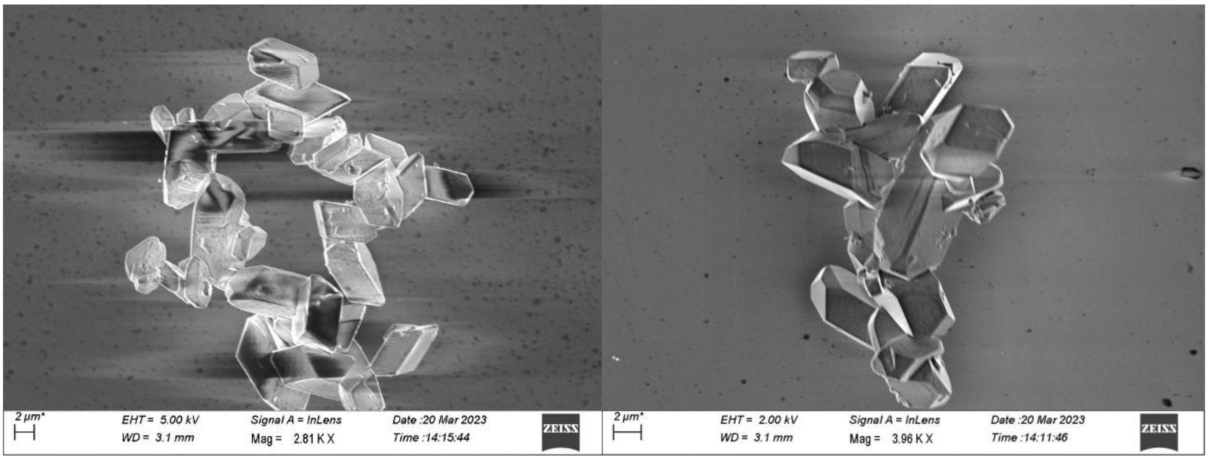

Figure S2 SEM images of UOW-10 crystals

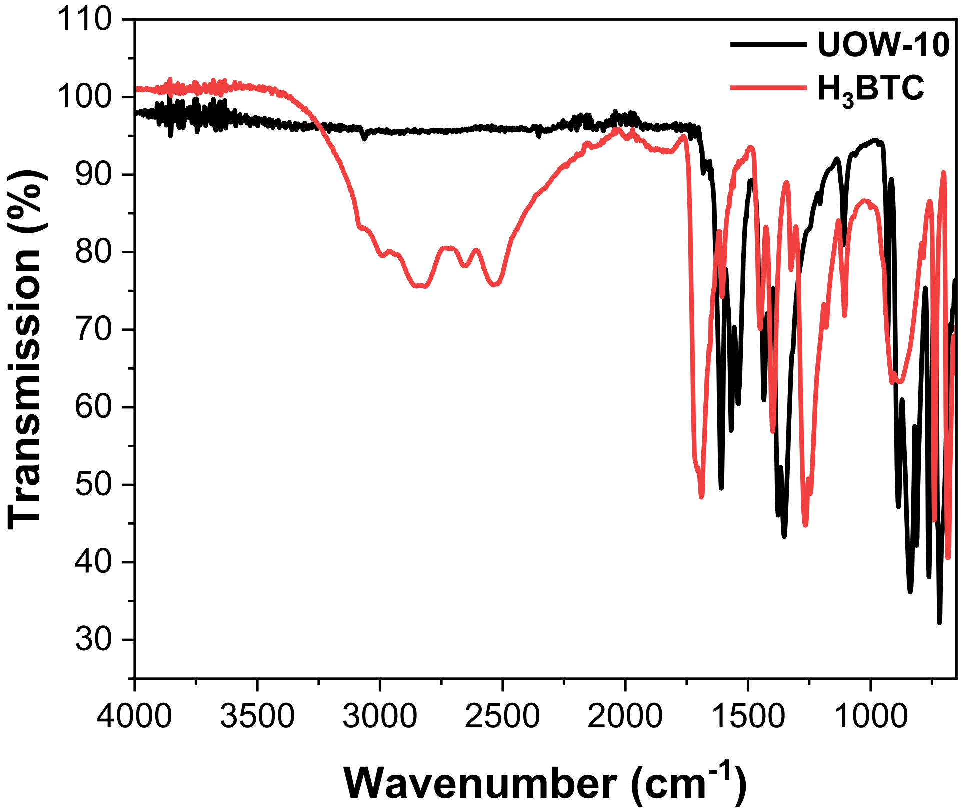

Figure S3 FT-IR spectra of UOW-10 and benzene-1,3,5-tricarboxylic acid (H_3BTC)

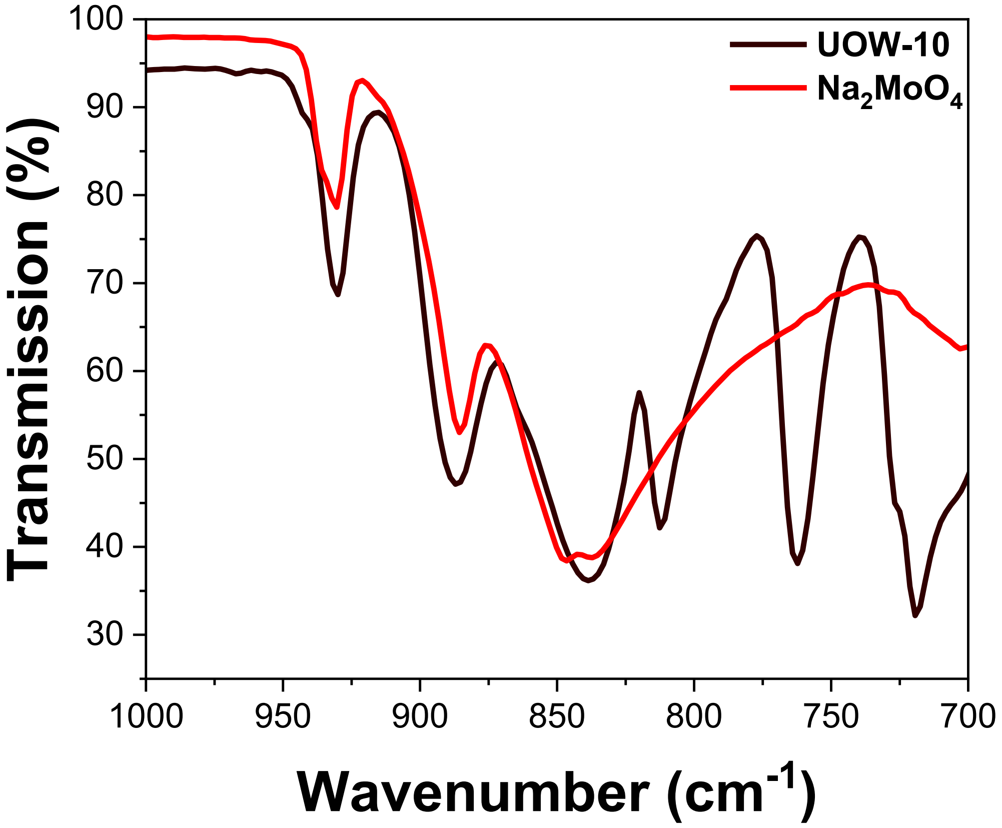

Figure S4 FT-IR spectra of UOW-10 and sodium molybdate (Na_2MoO_4)

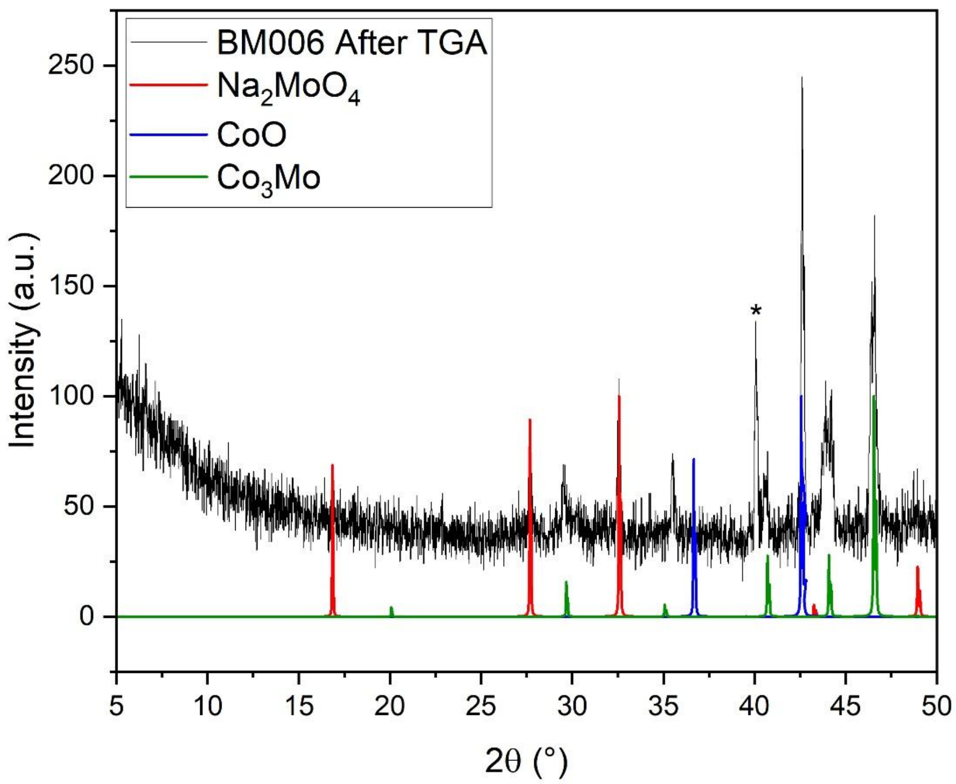

Figure S5 PXRD pattern of UOW-10 after TGA * indicates an unidentified Bragg peak.

Table S1 Bond lengths and metal centre separations of UOW-10 from 3D ED. Symmetry codes indicated by superscript.

**Atom1**	**Atom2**	**Separation (Å)**	**Atom1**	**Atom2**	**Separation (Å)**
Mo1	O1	1.709(8)	Co1	Na2	3.013(3)
Mo1	O2	1.699(10)	Co1	Na3[6]	2.968(3)
Mo1	O3	1.731(7)	Co1	Na4[3]	3.175(8)
Mo1	O4	1.705(7)	Mo1	Na1[1]	3.709(8)
Co1	O1[1]	2.090(7)	Mo1	Na2[2]	3.6034(19)
Co1	O3	1.976(7)	Mo1	Na3[7]	3.6269(19)
Co1	O4[2]	2.056(7)	Mo1	Na4[12]	3.465(9)
Co1	O6	2.008(8)	Mo1	Na4[3]	3.616(9)
Co1	O10[3]	2.100(10)	Na1	Na1[1]	3.4494(7)
Na1	O2[2]	2.438(12)	Na1	Na1[2]	3.4494
Na1	O2	2.538(12)	Na3	Na2[9]	3.4446
Na1	O5[2]	2.457(11)	Na3	Na2[1]	3.4446
Na1	O5	2.623(11)	Na4	Na4[1]	3.571(4)
Na1	O8[4]	2.540(14)	Na4	Na4[2]	3.571(4)
Na1	O8[5]	2.346(12)	C1	C2	1.382(11)
Na2	O1[1]	2.343(7)	C1	C6	1.409(13)
Na2	O1[6]	2.343(7)	C1	C7	1.476(13)
Na2	O6[7]	2.321(7)	C3	C2	1.349(13)
Na2	O9[3]	2.316(9)	C3	C4	1.399(13)
Na2	O9[8]	2.316(9)	C3	C8	1.486(12)
Na3	O4	1.234(11)	C4	C5	1.350(12)
Na3	O6[1]	2.390(7)	C5	C6	1.372(13)
Na3	O6[9]	2.390(7)	C5	C9	1.496(13)
Na3	O9[10]	2.339(9)	C7	O5	1.184(11)
Na3	O9[4]	2.339(9)	C7	O6	1.207(12)

Na4	O1^{1}	2.909(12)	C8	O7	1.250(13)
Na4	O3^{11}	2.490(11)	C8	O8	1.193(12)
Na4	O3^{1}	2.869(12)	C9	O9	2.362(7)
Na4	O7^{4}	2.269(12)	C9	O10	1.280(12)
Na4	O7^{8}	2.203(12)			
Na4	O8^{4}	2.815(14)			
Na4	O10	2.353(11)			

Symmetry codes: (1) +*x*,1.5−*y*,−0.5+*z*; (2) +*x*,1.5−*y*,0.5+*z*; (3) 1+*x*,+*y*,+*z*; (4) 1−*x*,0.5+*y*,1.5−*z*; (5) 1−*x*,1−*y*,2−*z*; (6) 2−*x*,−0.5+*y*,1.5−*z*; (7) 2−*x*,1−*y*,1−*z*; (8) 1−*x*,1−*y*,1−*z*; (9) 2−*x*,0.5+*y*,1.5−*z*; (10) 1+*x*,1.5−*y*,−0.5+*z*; (11) −1+*x*,+*y*,+*z*; (12) 1+*x*,1.5−*y*,0.5+*z*.

Table S2 Bond angles of UOW-10 from 3D ED. Symmetry codes indicated by superscript.

**Atom1**	**Atom2**	**Atom3**	**Angle (°)**	**Atom1**	**Atom2**	**Atom3**	**Angle (°)**
Na1^{1}	Mo1	O4	91.8(3)	O6^{12}	Na3	Mo1	94.56(17)
Na4^{2}	Mo1	O4	113.3(3)	O6^{1}	Na3	Mo1	85.44(17)
Na4^{3}	Mo1	O4	75.3(3)	O6^{12}	Na3	Mo1^{4}	85.44(17)
Na4^{3}	Mo1	Na1^{1}	104.62(18)	O6^{12}	Na3	Co1^{12}	42.33(18)
Na4^{2}	Mo1	Na1^{1}	143.55(19)	O6^{1}	Na3	Co1^{1}	42.33(18)
Na3^{4}	Mo1	O4	30.3(2)	O6^{1}	Na3	Co1^{12}	137.67(18)
Na3^{4}	Mo1	Na1^{1}	97.01(13)	O6^{12}	Na3	Co1^{1}	137.67(18)
Na3^{4}	Mo1	Na4^{2}	118.12(16)	O6^{12}	Na3	O4^{4}	73.6(3)
Na3^{4}	Mo1	Na4^{3}	102.75(13)	O6^{1}	Na3	O4^{4}	106.4(3)
Na2^{5}	Mo1	O4	84.5(2)	O6^{12}	Na3	O4	106.4(3)
Na2^{5}	Mo1	Na1^{1}	123.50(13)	O6^{1}	Na3	O4	73.6(3)
Na2^{5}	Mo1	Na4^{3}	128.18(15)	O6^{1}	Na3	O6^{12}	180.0
Na2^{5}	Mo1	Na4^{2}	86.42(14)	Na2^{12}	Na3	Mo1	61.21(3)
Na2^{5}	Mo1	Na3^{4}	56.90(3)	Na2^{12}	Na3	Mo1^{4}	118.79(3)
O3	Mo1	O4	111.2(4)	Na2^{13}	Na3	Mo1^{4}	61.21(3)
O3	Mo1	Na1^{1}	91.5(3)	Na2^{13}	Na3	Mo1	118.79(3)

O3	Mo1	Na4^{3}	38.0(3)	Na2^{13}	Na3	Co1^{12}	124.54(5)
O3	Mo1	Na4^{2}	55.6(3)	Na2^{12}	Na3	Co1^{1}	124.54(5)
O3	Mo1	Na3^{4}	140.4(3)	Na2^{13}	Na3	Co1^{1}	55.46(5)
O3	Mo1	Na2^{5}	141.9(3)	Na2^{12}	Na3	Co1^{12}	55.46(5)
O2	Mo1	O4	110.1(5)	Na2^{13}	Na3	O4	99.10(18)
O2	Mo1	Na1^{1}	31.9(3)	Na2^{12}	Na3	O4^{4}	99.10(18)
O2	Mo1	Na4^{3}	133.4(3)	Na2^{13}	Na3	O4^{4}	80.90(18)
O2	Mo1	Na4^{2}	136.6(4)	Na2^{12}	Na3	O4	80.90(18)
O2	Mo1	Na3^{4}	99.7(3)	Na2^{13}	Na3	O6^{1}	43.22(18)
O2	Mo1	Na2^{5}	98.2(3)	Na2^{12}	Na3	O6^{1}	136.78(18)
O2	Mo1	O3	107.4(4)	Na2^{12}	Na3	O6^{12}	43.22(18)
O1	Mo1	O4	107.5(4)	Na2^{13}	Na3	O6^{12}	136.78(18)
O1	Mo1	Na1^{1}	141.7(3)	Na2^{12}	Na3	Na2^{13}	180.0
O1	Mo1	Na4^{2}	56.9(3)	O9^{14}	Na3	Mo1^{4}	74.2(2)
O1	Mo1	Na4^{3}	112.1(3)	O9^{7}	Na3	Mo1^{4}	105.8(2)
O1	Mo1	Na3^{4}	85.6(2)	O9^{14}	Na3	Mo1	105.8(2)
O1	Mo1	Na2^{5}	32.3(2)	O9^{7}	Na3	Mo1	74.2(2)
O1	Mo1	O3	110.7(4)	O9^{14}	Na3	Co1^{12}	126.9(2)
O1	Mo1	O2	109.8(4)	O9^{7}	Na3	Co1^{12}	53.1(2)
Na4^{3}	Co1	O4^{5}	122.0(3)	O9^{7}	Na3	Co1^{1}	126.9(2)
Na3^{6}	Co1	O4^{5}	51.2(2)	O9^{14}	Na3	Co1^{1}	53.1(2)
Na3^{6}	Co1	Na4^{3}	141.2(2)	O9^{14}	Na3	O4	84.8(3)
O6	Co1	O4^{5}	88.0(3)	O9^{7}	Na3	O4^{4}	84.8(3)
O6	Co1	Na4^{3}	149.7(3)	O9^{7}	Na3	O4	95.2(3)
O6	Co1	Na3^{6}	53.2(2)	O9^{14}	Na3	O4^{4}	95.2(3)
Na2	Co1	O4^{5}	121.5(2)	O9^{7}	Na3	O6^{1}	105.9(3)
Na2	Co1	Na4^{3}	102.91(16)	O9^{14}	Na3	O6^{1}	74.1(3)
Na2	Co1	Na3^{6}	70.31(5)	O9^{7}	Na3	O6^{12}	74.1(3)
Na2	Co1	O6	51.5(2)	O9^{14}	Na3	O6^{12}	105.9(3)

O3	Co1	$O4^{5}$	95.3(3)	$O9^{7}$	Na3	$Na2^{13}$	138.0(2)
O3	Co1	$Na4^{3}$	51.7(3)	$O9^{7}$	Na3	$Na2^{12}$	42.0(2)
O3	Co1	$Na3^{6}$	146.5(3)	$O9^{14}$	Na3	$Na2^{12}$	138.0(2)
O3	Co1	O6	137.6(3)	$O9^{14}$	Na3	$Na2^{13}$	42.0(2)
O3	Co1	Na2	143.2(3)	$O9^{14}$	Na3	$O9^{7}$	180.0
$O10^{3}$	Co1	$O4^{5}$	92.4(4)	$Na3^{6}$	O6	Co1	84.4(3)
$O10^{3}$	Co1	$Na4^{3}$	47.8(3)	Na2	O6	Co1	86.8(3)
$O10^{3}$	Co1	$Na3^{6}$	93.6(2)	Na2	O6	$Na3^{6}$	92.9(3)
$O10^{3}$	Co1	O6	134.9(3)	C7	O6	Co1	114.2(6)
$O10^{3}$	Co1	Na2	91.8(3)	C7	O6	$Na3^{6}$	125.8(7)
$O10^{3}$	Co1	O3	87.3(3)	C7	O6	Na2	136.0(7)
$O1^{1}$	Co1	$O4^{5}$	172.3(3)	$Mo1^{6}$	Na2	$Mo1^{1}$	180.0
$O1^{1}$	Co1	$Na4^{3}$	63.2(3)	Co1	Na2	$Mo1^{1}$	64.64(6)
$O1^{1}$	Co1	$Na3^{6}$	121.1(2)	$Co1^{15}$	Na2	$Mo1^{6}$	64.64(6)
$O1^{1}$	Co1	O6	86.6(3)	$Co1^{15}$	Na2	$Mo1^{1}$	115.36(6)
$O1^{1}$	Co1	Na2	50.8(2)	Co1	Na2	$Mo1^{6}$	115.36(6)
$O1^{1}$	Co1	O3	92.4(3)	$Co1^{15}$	Na2	Co1	180.0
$O1^{1}$	Co1	$O10^{3}$	87.6(4)	$Na3^{6}$	Na2	$Mo1^{6}$	61.89(3)
$Co1^{1}$	O4	Mo1	140.8(4)	$Na3^{16}$	Na2	$Mo1^{1}$	61.89(3)
$Na3^{4}$	O4	Mo1	127.9(4)	$Na3^{6}$	Na2	$Mo1^{1}$	118.11(3)
$Na3^{4}$	O4	$Co1^{1}$	85.2(3)	$Na3^{16}$	Na2	$Mo1^{6}$	118.11(3)
$Na1^{5}$	Na1	$Mo1^{5}$	64.54(13)	$Na3^{6}$	Na2	$Co1^{15}$	125.77(5)
$Na1^{1}$	Na1	$Mo1^{5}$	117.82(16)	$Na3^{16}$	Na2	Co1	125.77(5)
$O2^{5}$	Na1	$Mo1^{5}$	21.6(2)	$Na3^{16}$	Na2	$Co1^{15}$	54.23(5)
O2	Na1	$Mo1^{5}$	92.1(3)	$Na3^{6}$	Na2	Co1	54.23(5)
$O2^{5}$	Na1	$Na1^{1}$	136.4(3)	$Na3^{6}$	Na2	$Na3^{16}$	180.0
O2	Na1	$Na1^{1}$	44.9(3)	$O6^{15}$	Na2	$Mo1^{6}$	84.34(17)
$O8^{7}$	Na1	$Mo1^{5}$	159.2(3)	O6	Na2	$Mo1^{1}$	84.34(17)
$O8^{8}$	Na1	$Mo1^{5}$	94.8(4)	$O6^{15}$	Na2	$Mo1^{1}$	95.66(17)

$O8^{7}$	Na1	$Na1^{1}$	42.8(3)	O6	Na2	$Mo1^{6}$	95.66(17)
$O8^{8}$	Na1	$Na1^{1}$	135.5(4)	$O6^{15}$	Na2	Co1	138.28(18)
$O8^{7}$	Na1	O2	68.4(3)	O6	Na2	Co1	41.72(18)
$O8^{7}$	Na1	$O2^{5}$	177.0(5)	O6	Na2	$Co1^{15}$	138.28(18)
$O8^{8}$	Na1	$O2^{5}$	73.2(4)	$O6^{15}$	Na2	$Co1^{15}$	41.72(18)
$O8^{8}$	Na1	O2	170.1(5)	O6	Na2	$Na3^{6}$	43.86(17)
$O5^{5}$	Na1	$Mo1^{5}$	73.5(3)	$O6^{15}$	Na2	$Na3^{6}$	136.14(17)
O5	Na1	$Mo1^{5}$	80.6(3)	O6	Na2	$Na3^{16}$	136.14(17)
O5	Na1	$Na1^{1}$	49.3(3)	$O6^{15}$	Na2	$Na3^{16}$	43.86(17)
$O5^{5}$	Na1	$Na1^{1}$	129.1(4)	$O6^{15}$	Na2	O6	180.0
O5	Na1	O2	72.6(3)	$O9^{11}$	Na2	$Mo1^{6}$	105.0(2)
O5	Na1	$O2^{5}$	91.6(4)	$O9^{11}$	Na2	$Mo1^{1}$	75.0(2)
$O5^{5}$	Na1	O2	88.2(4)	$O9^{3}$	Na2	$Mo1^{1}$	105.0(2)
$O5^{5}$	Na1	$O2^{5}$	71.4(3)	$O9^{3}$	Na2	$Mo1^{6}$	75.0(2)
O5	Na1	$O8^{7}$	86.5(4)	$O9^{3}$	Na2	Co1	52.5(2)
$O5^{5}$	Na1	$O8^{7}$	111.4(4)	$O9^{11}$	Na2	$Co1^{15}$	52.5(2)
$O5^{5}$	Na1	$O8^{8}$	86.9(4)	$O9^{3}$	Na2	$Co1^{15}$	127.5(2)
O5	Na1	$O8^{8}$	115.6(4)	$O9^{11}$	Na2	Co1	127.5(2)
$Co1^{9}$	Na4	$Mo1^{9}$	62.85(17)	$O9^{3}$	Na2	$Na3^{16}$	137.5(2)
$Co1^{9}$	Na4	$Mo1^{10}$	64.93(18)	$O9^{3}$	Na2	$Na3^{6}$	42.5(2)
$Na4^{5}$	Na4	$Mo1^{10}$	127.70(14)	$O9^{11}$	Na2	$Na3^{6}$	137.5(2)
$Na4^{1}$	Na4	$Mo1^{9}$	105.3(2)	$O9^{11}$	Na2	$Na3^{16}$	42.5(2)
$Na4^{5}$	Na4	$Mo1^{9}$	57.65(17)	$O9^{3}$	Na2	$O6^{15}$	104.9(3)
$Na4^{1}$	Na4	$Mo1^{10}$	61.83(14)	$O9^{11}$	Na2	$O6^{15}$	75.1(3)
$Na4^{5}$	Na4	$Co1^{9}$	71.28(18)	$O9^{3}$	Na2	O6	75.1(3)
$Na4^{1}$	Na4	$Co1^{9}$	126.7(2)	$O9^{11}$	Na2	O6	104.9(3)
$O3^{9}$	Na4	$Mo1^{10}$	74.9(3)	$O9^{11}$	Na2	$O9^{3}$	180.0
$O3^{10}$	Na4	$Mo1^{9}$	69.4(3)	$O1^{1}$	Na2	$Mo1^{1}$	22.91(19)
$O3^{10}$	Na4	$Mo1^{10}$	29.87(16)	$O1^{1}$	Na2	$Mo1^{6}$	157.09(19)

$O3^{9}$	Na4	$Mo1^{9}$	25.32(19)	$O1^{6}$	Na2	$Mo1^{1}$	157.09(19)
$O3^{9}$	Na4	$Co1^{9}$	38.50(19)	$O1^{6}$	Na2	$Mo1^{6}$	22.91(19)
$O3^{10}$	Na4	$Co1^{9}$	87.7(3)	$O1^{6}$	Na2	Co1	136.25(18)
$O3^{9}$	Na4	$Na4^{1}$	122.8(3)	$O1^{1}$	Na2	$Co1^{15}$	136.25(18)
$O3^{10}$	Na4	$Na4^{1}$	43.8(2)	$O1^{6}$	Na2	$Co1^{15}$	43.75(18)
$C8^{7}$	Na4	$Mo1^{10}$	146.5(4)	$O1^{1}$	Na2	Co1	43.75(18)
$C8^{7}$	Na4	$Mo1^{9}$	84.9(3)	$O1^{6}$	Na2	$Na3^{6}$	82.03(17)
$C8^{7}$	Na4	$Co1^{9}$	129.9(3)	$O1^{6}$	Na2	$Na3^{16}$	97.97(17)
$C8^{7}$	Na4	$Na4^{1}$	96.9(3)	$O1^{1}$	Na2	$Na3^{6}$	97.97(17)
$C8^{7}$	Na4	$O3^{9}$	100.1(4)	$O1^{1}$	Na2	$Na3^{16}$	82.03(17)
$C8^{7}$	Na4	$O3^{10}$	117.0(4)	$O1^{1}$	Na2	O6	73.4(2)
$O8^{7}$	Na4	$Mo1^{9}$	109.4(3)	$O1^{6}$	Na2	$O6^{15}$	73.4(2)
$O8^{7}$	Na4	$Mo1^{10}$	152.6(4)	$O1^{1}$	Na2	$O6^{15}$	106.6(2)
$O8^{7}$	Na4	$Co1^{9}$	142.3(4)	$O1^{6}$	Na2	O6	106.6(2)
$O8^{7}$	Na4	$Na4^{1}$	90.9(3)	$O1^{6}$	Na2	$O9^{3}$	97.3(3)
$O8^{7}$	Na4	$O3^{10}$	125.9(4)	$O1^{1}$	Na2	$O9^{3}$	82.7(3)
$O8^{7}$	Na4	$O3^{9}$	122.6(4)	$O1^{1}$	Na2	$O9^{11}$	97.3(3)
$O8^{7}$	Na4	$C8^{7}$	24.6(3)	$O1^{6}$	Na2	$O9^{11}$	82.7(3)
$O7^{7}$	Na4	$Mo1^{9}$	62.9(3)	$O1^{1}$	Na2	$O1^{6}$	180.0
$O7^{7}$	Na4	$Mo1^{10}$	137.9(5)	Co1	O3	Mo1	147.6(5)
$O7^{11}$	Na4	$Mo1^{10}$	66.4(3)	$Na4^{2}$	O3	Mo1	94.5(3)
$O7^{11}$	Na4	$Mo1^{9}$	137.3(5)	$Na4^{3}$	O3	Mo1	116.7(4)
$O7^{11}$	Na4	$Co1^{9}$	116.0(4)	$Na4^{2}$	O3	Co1	107.5(3)
$O7^{7}$	Na4	$Co1^{9}$	104.8(3)	$Na4^{3}$	O3	Co1	89.8(3)
$O7^{11}$	Na4	$Na4^{1}$	37.7(3)	$Na2^{9}$	O9	$Na3^{17}$	95.5(3)
$O7^{7}$	Na4	$Na4^{1}$	114.7(5)	C9	O9	$Na3^{17}$	130.3(7)
$O7^{7}$	Na4	$O3^{10}$	115.9(4)	C9	O9	$Na2^{9}$	124.2(7)
$O7^{11}$	Na4	$O3^{9}$	141.1(5)	Na1	O2	Mo1	128.1(5)
$O7^{7}$	Na4	$O3^{9}$	74.5(4)	$Na1^{1}$	O2	Mo1	126.5(5)

O7[11]	Na4	O3[10]	67.9(4)	O8	C8	Na4[17]	78.6(7)
O7[11]	Na4	C8[7]	113.7(4)	C3	C8	Na4[17]	144.2(7)
O7[7]	Na4	C8[7]	26.0(3)	C3	C8	O8	121.4(9)
O7[11]	Na4	O8[7]	94.7(4)	O7	C8	Na4[17]	52.8(6)
O7[7]	Na4	O8[7]	48.4(3)	O7	C8	O8	122.7(9)
O10	Na4	Mo1[9]	95.7(3)	O7	C8	C3	115.9(8)
O10	Na4	Mo1[10]	95.4(4)	Na4[17]	O8	Na1[8]	105.5(4)
O10	Na4	Co1[9]	41.4(3)	Na4[17]	O8	Na1[17]	114.5(4)
O10	Na4	Na4[1]	143.5(5)	C8	O8	Na1[17]	109.4(9)
O10	Na4	O3[10]	124.1(4)	C8	O8	Na1[8]	158.0(9)
O10	Na4	O3[9]	71.0(3)	C8	O8	Na4[17]	76.9(7)
O10	Na4	C8[7]	114.6(4)	C2	C3	C8	121.4(9)
O10	Na4	O8[7]	110.0(5)	C4	C3	C8	119.1(8)
O10	Na4	O7[11]	108.8(5)	C4	C3	C2	119.4(8)
O10	Na4	O7[7]	101.3(4)	C8	O7	Na4[11]	134.5(8)
O1[10]	Na4	Mo1[10]	29.48(17)	C8	O7	Na4[17]	101.2(7)
O1[10]	Na4	Mo1[9]	80.1(3)	Na4	O10	Co1[9]	90.8(4)
O1[10]	Na4	Co1[9]	39.89(18)	C9	O10	Co1[9]	98.0(7)
O1[10]	Na4	Na4[1]	88.4(2)	C9	O10	Na4	137.6(8)
O1[10]	Na4	O3[9]	65.4(3)	C2	C1	C7	122.6(8)
O1[10]	Na4	O3[10]	58.6(3)	C6	C1	C7	119.0(8)
O1[10]	Na4	C8[7]	164.9(4)	C6	C1	C2	118.5(8)
O1[10]	Na4	O8[7]	170.2(4)	C1	C7	O6	115.9(7)
O1[10]	Na4	O7[11]	78.9(4)	O5	C7	O6	121.3(9)
O1[10]	Na4	O7[7]	139.9(5)	O5	C7	C1	122.7(9)
O1[10]	Na4	O10	65.9(3)	C1	C2	C3	121.4(9)
Mo1	Na3	Mo1[4]	180.0	C5	C4	C3	120.4(9)
Co1[1]	Na3	Mo1	64.11(6)	C7	O5	Na1	134.7(8)
Co1[12]	Na3	Mo1	115.89(6)	C7	O5	Na1[1]	134.9(8)

$Co1^{12}$	Na3	$Mo1^{4}$	64.11(6)	C6	C5	C4	120.8(9)
$Co1^{1}$	Na3	$Mo1^{4}$	115.89(6)	C9	C5	C4	121.7(9)
$Co1^{1}$	Na3	$Co1^{12}$	180.0	C9	C5	C6	117.3(8)
O4	Na3	Mo1	21.77(18)	C5	C6	C1	119.5(8)
$O4^{4}$	Na3	Mo1	158.23(18)	O10	C9	O9	123.2(10)
O4	Na3	$Mo1^{4}$	158.23(18)	C5	C9	O9	119.2(9)
$O4^{4}$	Na3	$Mo1^{4}$	21.77(18)	C5	C9	O10	117.6(9)
O4	Na3	$Co1^{12}$	136.35(18)	$Co1^{5}$	O1	Mo1	140.0(4)
$O4^{4}$	Na3	$Co1^{1}$	136.35(18)	$Na4^{2}$	O1	Mo1	93.6(3)
O4	Na3	$Co1^{1}$	43.65(18)	$Na4^{2}$	O1	$Co1^{5}$	76.9(3)
$O4^{4}$	Na3	$Co1^{12}$	43.65(18)	$Na2^{5}$	O1	Mo1	124.8(4)
$O4^{4}$	Na3	O4	180.0	$Na2^{5}$	O1	$Co1^{5}$	85.4(3)
$O6^{1}$	Na3	$Mo1^{4}$	94.56(17)	$Na2^{5}$	O1	$Na4^{2}$	134.1(4)

Symmetry Codes: (1) +*x*,3/2-*y*,-1/2+*z*; (2) 1+*x*,3/2-*y*,1/2+*z*; (3) 1+*x*,+*y*,+*z*; (4) 2-*x*,2-*y*,1-*z*; (5) +*x*,3/2-*y*,1/2+*z*; (6) 2-*x*,-1/2+*y*,3/2-*z*; (7) 1-*x*,1/2+*y*,3/2-*z*; (8) 1-*x*,1-*y*,2-*z*; (9) -1+*x*,+*y*,+*z*; (10) -1+*x*,3/2-*y*,-1/2+*z*; (11) 1-*x*,1-*y*,1-*z*; (12) 2-*x*,1/2+*y*,3/2-*z*; (13) 2-*x*,1/2+*y*,1/2-*z*; (14) 1+*x*,3/2-*y*,-1/2+*z*; (15) 2-*x*,1-*y*,1-*z*; (16) 2-*x*,-1/2+*y*,1/2-*z*; (17) 1-*x*,-1/2+*y*,3/2-*z*

Table S3 Bond valence sum calculation for cobalt

j	*r*0 (Å)	*r*ij (Å)	*B* (Å)	*S*ij	*V*i
1	1.692	2.090(7)	0.37	0.34	
2	1.692	1.976(7)	0.37	0.46	
3	1.692	2.056(7)	0.37	0.37	
4	1.692	2.008(8)	0.37	0.43	
5	1.692	2.100(10)	0.37	0.33	1.94

Table S4 Bond valence sum calculation for molybdenum

j	*r*0 (Å)	*r*ij (Å)	*B* (Å)	*S*ij	*V*i
1	1.872	1.705(7)	0.37	1.57	

2	1.872	1.731(7)	0.37	1.46	
3	1.872	1.709(8)	0.37	1.55	
4	1.872	1.699(10)	0.37	1.60	6.18

Table S5 Deconvolution of UOW-10 UV-Vis spectra

Model	Gaussian			
Equation	$y = y0 + \frac{A}{\left(w * \left(\sqrt{\frac{\pi}{4 * \ln(2)}}\right)\right)} * e^{\left(\frac{-4 * \ln(2) * (x - xc)^2}{w^2}\right)}$			
Plot	Peak 1	Peak 2	Peak 3	Peak 4
y0 (a.u.)	0.11228 ± 3.73037E-4	0.11228 ± 3.73037E-4	0.11228 ± 3.73037E-4	0.11228 ± 3.73037E-4
xc (nm)	508.48433 ± 0.18922	540.62083 ± 0.13569	572.75186 ± 0.3552	620.03739 ± 0.46781
A (nm)	29.37122 ± 0.22668	1.07517 ± 0.0736	21.60259 ± 0.80412	24.32457 ± 0.59781
w (nm)	65.39476 ± 0.25272	21.01901 ± 0.56093	57.6281 ± 1.23851	57.05039 ± 0.42522
Reduced Chi-Sqr	1.64872E-6			
R-Square (COD)	0.99994			
Adj. R-Square	0.99994			
